# In vivo immunogenicity assessment and vaccine efficacy evaluation of a chimeric tandem repeat of epitopic region of OMP31 antigen fused to interleukin 2 (IL-2) against *Brucella melitensis* in BALB/c mice

**DOI:** 10.1186/s12917-019-2074-7

**Published:** 2019-11-08

**Authors:** Narges Nazifi, Mojtaba Tahmoorespur, Mohammad Hadi Sekhavati, Alireza Haghparast, Ali Mohammad Behroozikhah

**Affiliations:** 1 Department of Animal Science, Faculty of Agriculture, Ferdowsi University of Mashhad, Mashhad, Iran; 2 Department of Pathobiology, Faculty of Veterinary Medicine, Ferdowsi University of Mashhad, Mashhad, Iran; 3 Department of Brucellosis, Razi Vaccine and Serum Research institute, agricultural Research Education and Extension Organization (AREEO), Karaj, Iran

**Keywords:** Brucellosis, *Brucella melitensis*, Experimental epitope, OMP31, Recombinant protein, Immunization, Cytokines, Antibodies, Adjuvant

## Abstract

**Background:**

Designing a potent recombinant vaccine, using the appropriate subunits with the greatest effect on stimulating the immune system, especially in the case of intracellular pathogens such as gram negative *Brucella Melitensis* bacteria, is of great importance. In this study, three repeats of 27 amino acids of the immunogenic epitope derived from OMP31 antigen (3E) from the *Brucella melitensis*, in a protective manner against Brucellosis have been used. To fortify the delivery system of recombinant antigens, IL-2 cytokine as a molecular adjuvant was fused to recombinant constructs. Recombinant proteins were evaluated for immunological studies in a mouse model (BALB/c).

**Results:**

The results showed that all recombinant proteins could stimulate the immune system to produce Th1 cytokines and antibodies in compare to the negative control treatments. 3E-IL2 and then OMP31-IL2 proteins stimulated higher levels of IFN-γ and IL-2 compared to the other treatments (*p* < 0.05). Also, the results indicated that experimental treatments produced a higher level of IgG2a isotype than IgG1 isotype. In addition, the findings of the experiment showed that the presence of chemical adjuvant (IFA) along with molecular adjuvant can play a significant role in stimulating the immune system. After determining the potency of recombinant structures, their efficacy in stimulating the immune system were also evaluated. *B. melitensis M16* strain was used to challenge 30 days after last immunization. The microbial load of the splenocyte in the treatments receiving chimeric proteins were significantly lower. Also, Wright serological test confirmed that these treatments had the lowest agglutination rate, as well as the positive treatment, while in the negative treatments in excess of blood serum dilutions, agglutination rate were more than 2 + .

**Conclusions:**

3E-IL2 treatment showed the best performance compared to other recombinant proteins and could be considered as the suitable candidate for further research on the production of recombinant vaccine against Brucella.

## Background

Brucellosis (Malt fever) known as a zoonotic disease which caused by gram negative *Brucella Melitensis* bacteria, as an intracellular pathogen in mammalians. Reduced fertility rates and milk production are the most common symptoms of this disease in livestock. The most common transmission way of this infection to human is using contaminated products [1]. Protective immunity against infection by Brucella spp. involves a cascade on immunity factors including the innate immunity, CD4+ and CD8+ T lymphocytes, macrophages (MΦ), dendritic cells (DCs) and inflammatory cytokines like IFN-γ and IFN α [1, 2]. So far live attenuated strains (e.g. Rev1) have been used to protect against the brucellosis but these vaccines cause abortion in pregnant animals. In addition, the current vaccines interfere in serological tests as well as are resistance to streptomycin and cause infection and disease in human [3]. Using recombinant vaccine as an inert vaccine offers advantages over whole organisms which not only can omit disadvantages of live vaccines but also introduce some advantages such as safety and purity. But these vaccines are not enough strong to stimulate strong immune responses [4] . Factors which should be considered in using inert vaccines are; selection an appropriate antigen and best adjuvant and also using a good delivery system [5]. OMP31, the 31 KDa outer membrane protein, known as protective antigen which used as DNA vaccine in high concentration against *B. melitensis* and *B. ovis* challenges [6]. It has been reported that, immunization of BALB/c mice with rOmp31 conferred a robust immunoglobulin G (IgG) response along with production the interleukin 2 (IL-2) and gamma interferon, but not IL-10 or IL-4, which refers to induction T helper 1 (Th1) response and also a good CTL (Cytotoxic-T-lymphocyte) response which related to induce the CD8 + T [7, 8]. Using OMP31 extract in immunity studies, also coffered both humoral and cellular immunity [9]. Previous studies demonstrated that an exposed and hydrophobic loop of OMP31 antigen, located between 43 and 83 amino acid residuals, is conserved among the different strain of Brucella spp., and is cognate with mAb (A59/10F09/G10) [10, 11]. A short peptide of 48–74 residues of Omp31 (Omp31_48–74_) is a T helper (Th) 1 response inducer that presents a proper protection against *B. melitensis* [8]. So far, studies have shown that the use of epitopes as subunit in immunogenes structures has been very successful in stimulating the immune system (cellular and humoral) to protect against Brucellosis [12, 13]. Genetic adjuvants related to some genes encode cytokines, chemokines, costimulatory factors and some other molecules which frequently are involved in co-administration with antigens to change the magnitude, duration and nature of immune response [14]. When the host encounters an antigen, evoking the immunity system is being done through the cellular mediated immunity procedures which act as an intracellular pathogen trapped inside the antigen-presenting cell (APCs) located in lymphoid organs. Then antigens being degraded and their peptides being presented to MHCI and MHCI markers which call T-lymphocytes contain CD8+ and CD + 4 markers, respectively [15]. Effector T cells produce IL-2 as an autocrine growth factor which leads to the differentiation of T cells into a specific lineages of T cell [16, 17].

In this study, we aimed to design some subunit vaccines including OMP31 antigen and its immunogenic epitopes in form of fusion to IL-2 as a molecular adjuvant, and investigating their desired potency and efficacy in stimulation the immune system.

## Results

### Expression of recombinant antigenic structures in prokaryote system

**Fig. 1:**
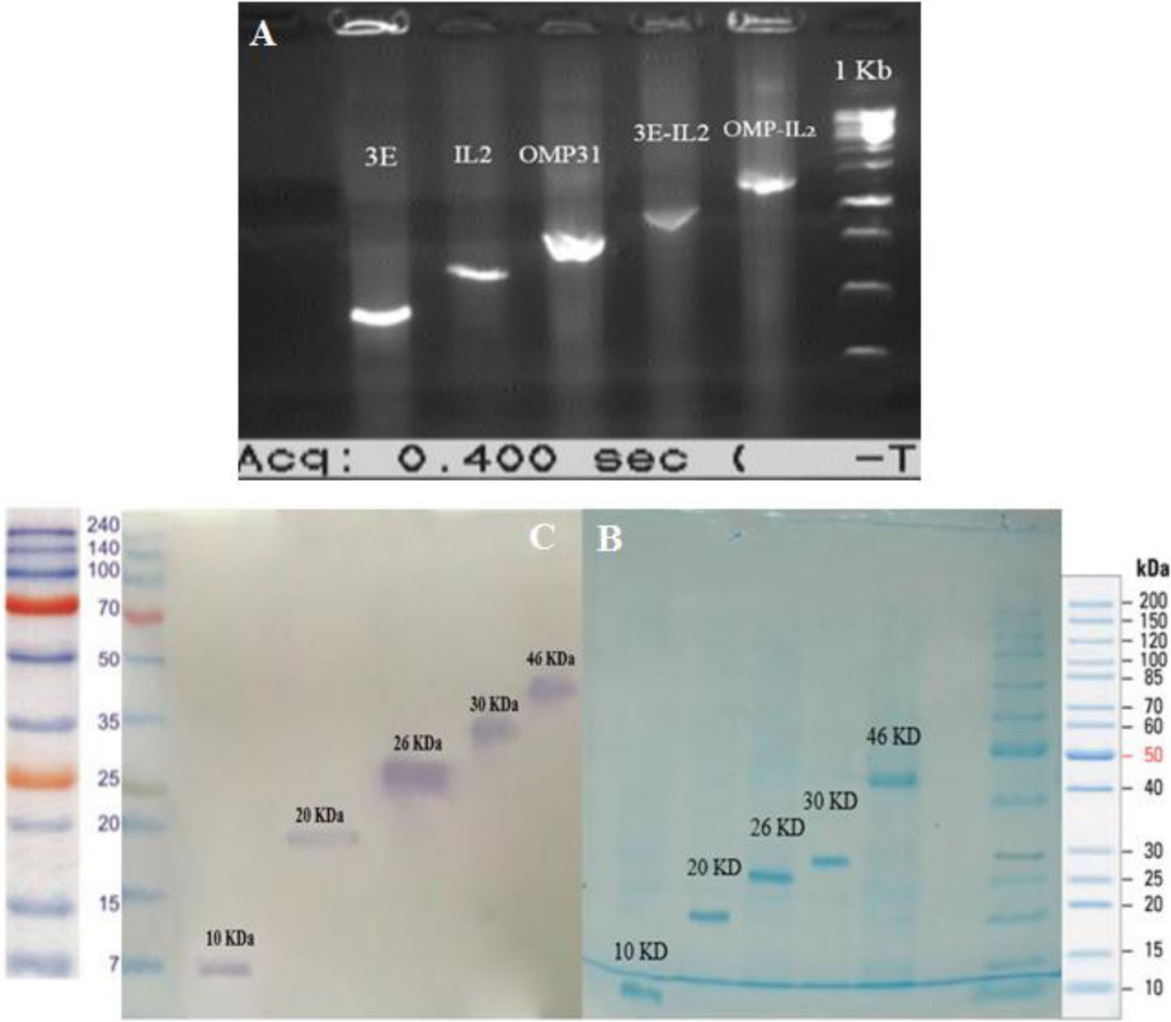
Preparation and confirmation of production the recombinant protein. **a** confirmation of inserting the recombinant gens into pET-22b (+) vector through colony-PCR process using T7 universal primer. **b** SDS-PAGE analysis which approves purification the recombinant proteins correctly using Ni-NTA column. **c** Western blot confirmation of recombinant proteins by Anti Poly-Histidine-HRP antibody

Each of univalent structures (OMP31, 3E and IL-2 gens) were successfully amplified using donated or synthesized vectors and were properly ligated into pTZ57R/T and then pET-22b (+) vector. OMP31-IL2 and 3E-IL2 constructions also have already been ligated into the PET-22b (+) vector [18, 19]. After verifying the integrity of these five recombinant structures by sequencing and colony-PCR process with T7 universal primers (Fig. 1a), these structures were successfully transmitted to the prokaryotic expressive system (BL21 (D3) bacteria) using heat-shock process. The BL21 bacteria containing the recombinant plasmids were cultured in the penicillin-containing 2XYP medium under overnight incubation and then were rejuvenated for two-hour in 2XYP medium. Expression induction of the target proteins was accomplished using 0.5 mM IPTG correctly. As shown in Fig. 1b, the process of purifying the proteins using the Ni-NTA affinity chromatography columns was successfully performed and confirmed by SDS-PAGE (12%) and western blotting assay (Fig. 1c). Quantity of each yielded protein was assessed using Bradford assay and demonstrated that estimated concentration of OMP31, 3E, IL2, OMP31-IL2 and 3E-IL2 proteins were 0.6, 0.3, 0.5, 0.7 and 0.7 g/L respectively (R^2^ = 0.989).

### Desired potency of recombinant immunogenic proteins in stimulation of cellular immune responses

Assessment of cellular immune responses was done through measuring the IFN- γ, IL-2 and IL-4 cytokines by ELISA in splenocytes of immunized mice. One of the main factors in inducing protective immunity against various types of intracellular infections is production the IFN-γ cytokine. In general, ELISA results showed that recombinant antigens significantly effected (*P* < 0.05) on induction of IFN-γ secretion.

**Fig. 2:**
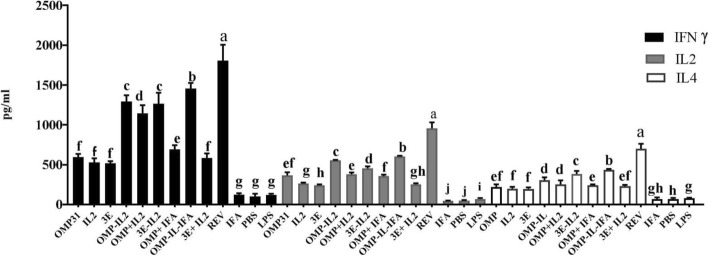
Determination of cytokine responses in splenocyte of immunized mice with different recombinant proteins. Levels of each cytokine were quantified (pg/mL) by ELISA. Mean comparison of treatments were carried out employing Tukey’s test with significance level of 0.05. Each value represents the mean of triplicates ± SD of cytokine responses from five samples. The experiment was replicated three times. Different letters indicate significantly different between experimental groups (*P* < .05)

OMP31-IL2 + IFA had the highest mean of IFN-γ, IL-2 and IL-4 (among the recombinant proteins), followed by 3E-IL2 protein and OMP31-IL2 protein without using IFA (*P* < 0.05) (Fig. 2). As can be seen in Fig. 2, statistically, cytokines are more secreted in treatments which received chimeric proteins than the treatments which received univalent or divalent recombinant proteins (*P* < 0.001). Moreover, as shown in Fig. 2, the proteins formulated in incomplete Freund's chemist adjuvant (IFA) in the two trials (OMP31 + IFA and OMP31-IL2 + IFA) were significantly different from those of the same but without formulation in the IFA adjuvant. Therefore, it can be argued that the use of chemical adjuvants along with the use of molecular adjuvants can boost the immune responses in a more positive effect.

### Immunization with chimeric recombinant immunogenic proteins represented to a desired humoral immune response

Humoral immune response against recombinant immunogenic proteins was evaluated by measuring the IgG antibody. In this study, the level of IgG titer was analyzed in three different conditions. For this purpose, the antibodies were measured at the end of 30 days after the last injection in eight different dilutions (1:50–1:64000) against the *B. melitensis* Rev1vaccine, OMP31 antigen and 3E construct.

Total IgG antibody titer showed that all experimental treatments produced higher anti-IgG antibodies compared to negative control groups at different concentrations of sera. The highest total IgG antibody titer related to the positive control group. The antigenic groups of Omp31-IL2 + IFA, 3E-IL2 and Omp31-IL2 showed the least variation with the positive control group respectively.

**Fig. 3:**
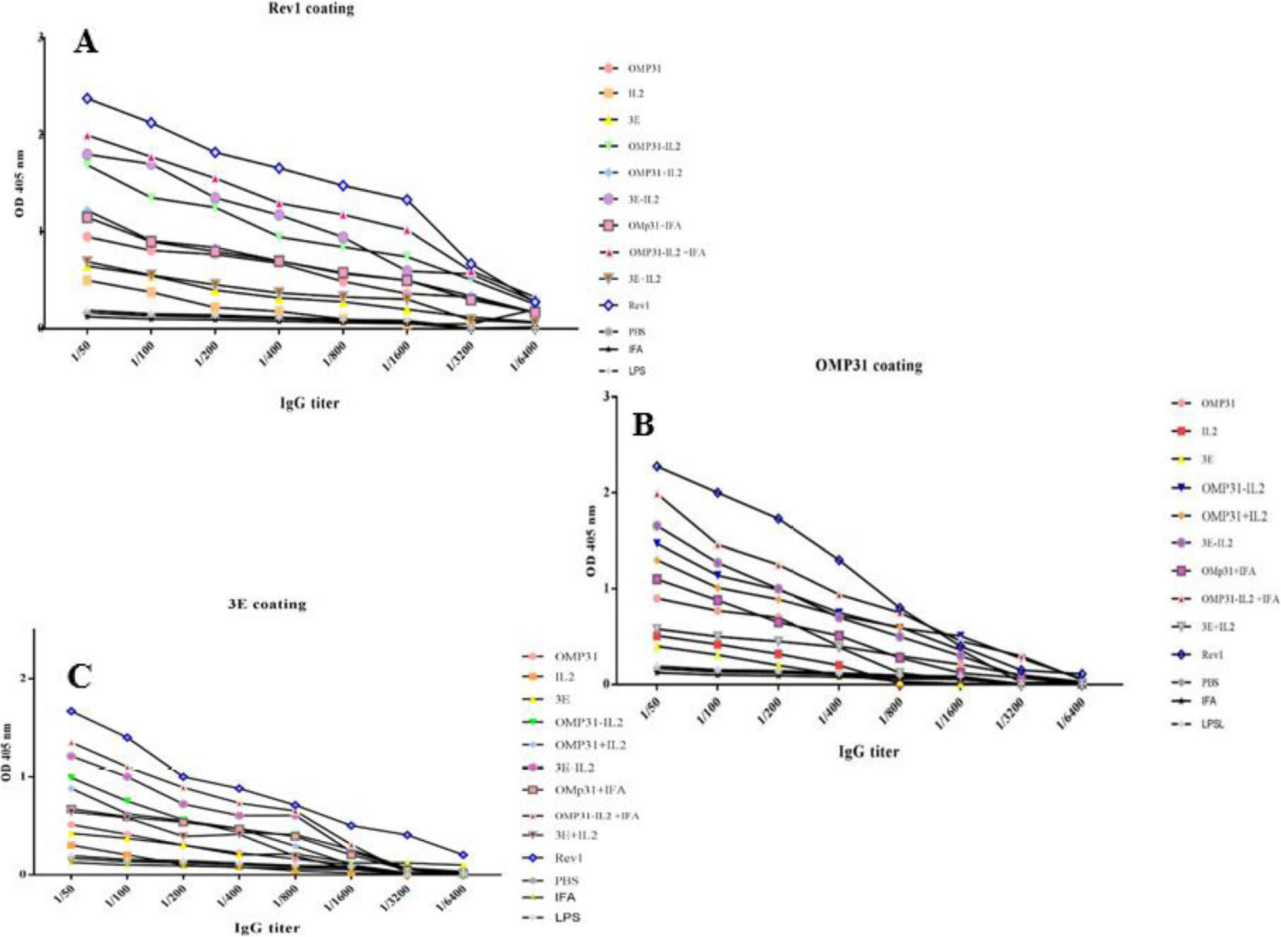
Titer of total IgG. Total IgG against RevI vaccine (**a**), rOmp31 protein (**b**) and r3E protein (**c**) in different dilution of sera (1/50–1/6400) at OD 405 nm

The IL-2 cytokine as a molecular adjuvant has improved the antigenic activity of the OMP31 antigen and the 3E construct (Fig. 3). So that, treatments which received chimera proteins showed better performance than those treated with univalent and divalent proteins (Fig. 3). On the other hand, formulation of proteins in chemical IFA compared to similar treatments without formulation in IFA (OMP31-IL2 + IFA compared to OMP31-IL2 and OMP31 + IFA compared with OMP31) improved stimulation the humoral immunity.

### Analysis of IgG1 and IgG2a isotypes responses after immunization

**Fig. 4:**
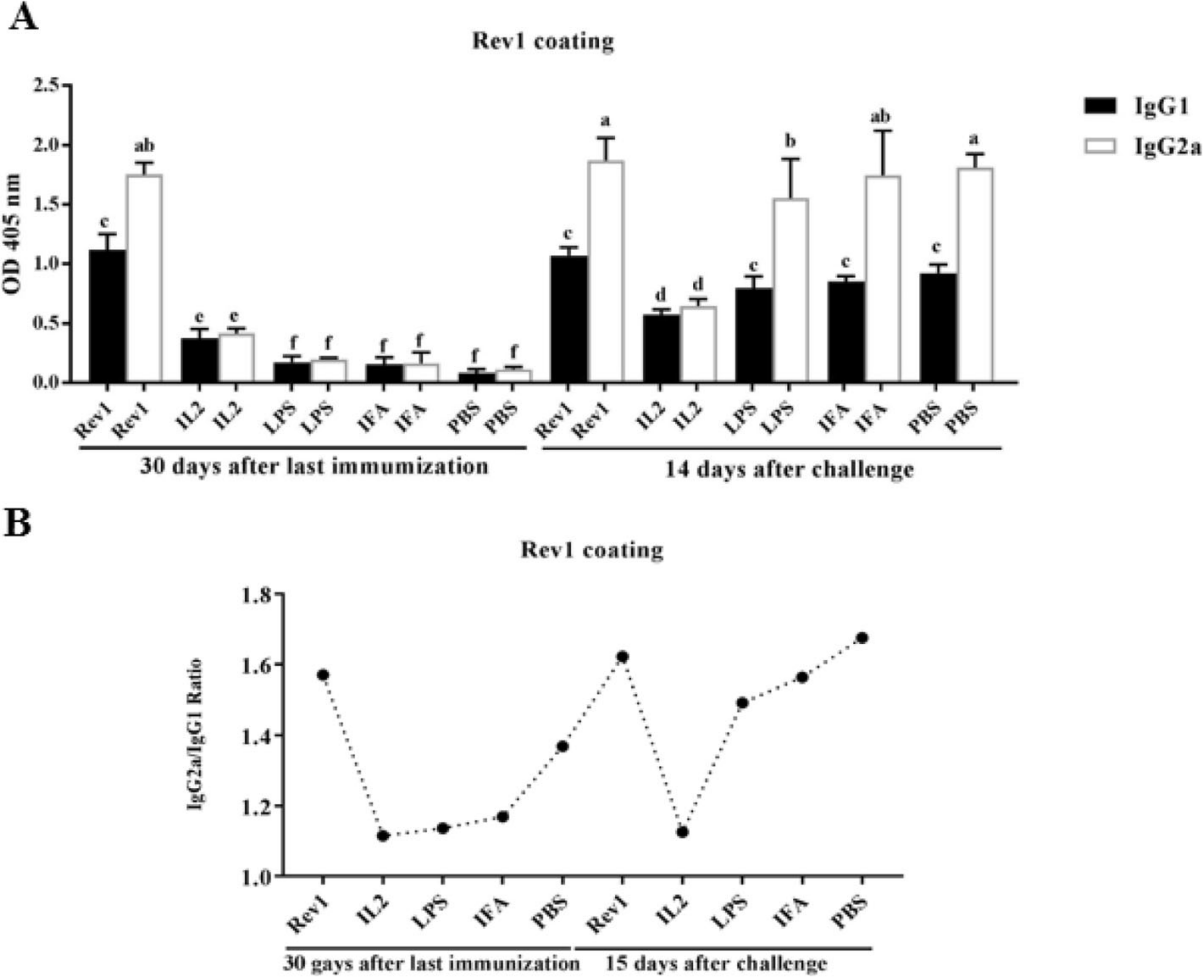
Titer of IgG1 and IgG2a isotypes against Rev1 vaccine**. a** the results of IgG1 and IgG2a isotypes titer comparison were represented in 1/50 dilution of sera against Rev1 vaccine. Mean comparison of treatments were carried out employing Tukey’s test with significance level of 0.05. **b** Ratio of IgG2a to IgG1 in different treatments. Levels of each antibody were measured at OD 405 nm by an ELISA reader. Each value represents the mean of triplicates ± SD of antibody responses from five samples. The experiment was replicated three times. Different letters are significantly different between experimental groups (*P* < .05)

**Fig. 5:**
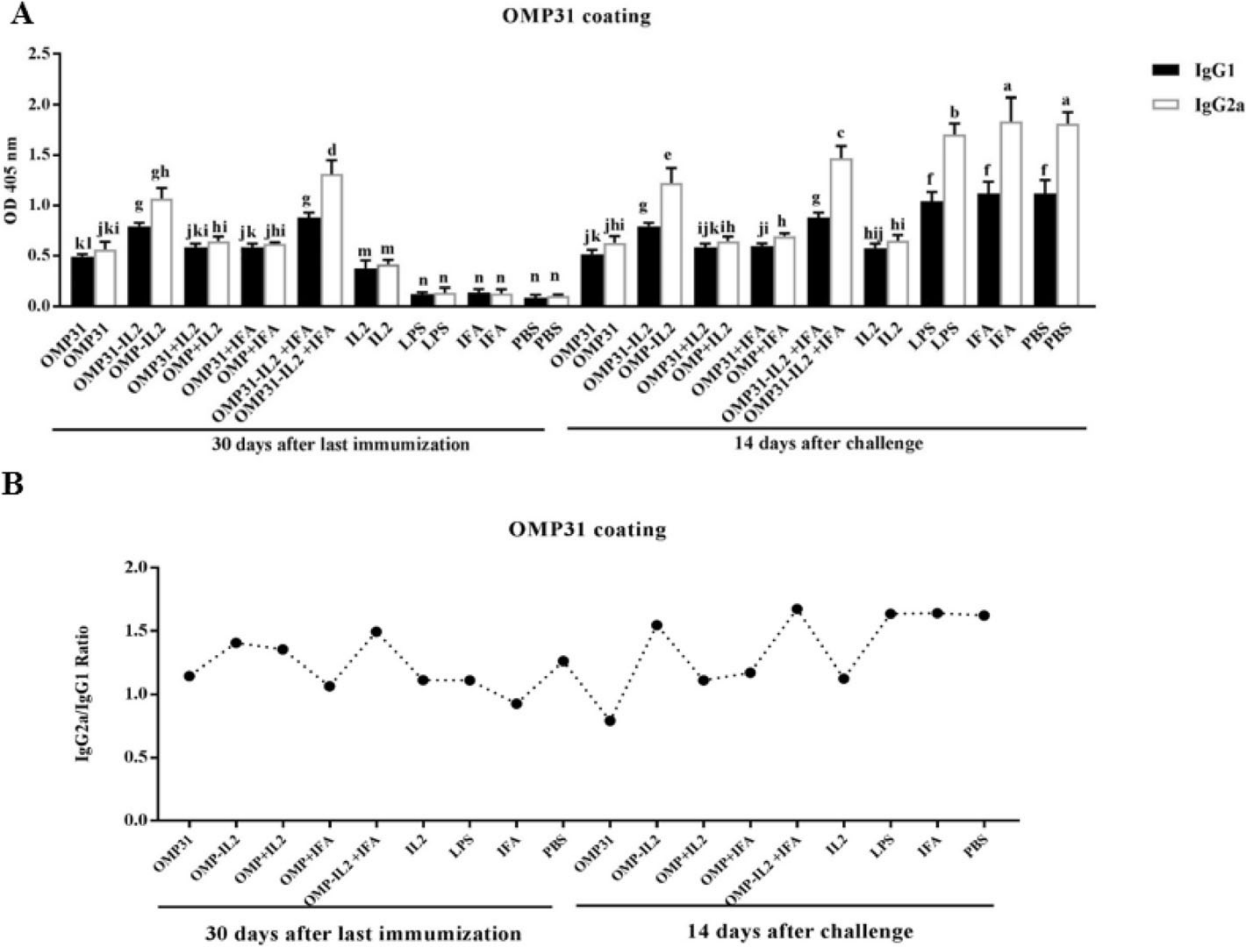
Titer of IgG1 and IgG2a isotypes against OMP31 protein. **a** the titer comparison of IgG1 and IgG2a isotypes related to 1/50 dilution of sera against OMP31 protein. Mean comparison of treatments, with significance level of 0.05, were carried out using Tukey’s test. **b** Ratio of IgG2a to IgG1 in different treatments. Levels of each antibody were measured at OD 405 nm by an ELISA reader. Each value represents the mean of triplicates ± SD of antibody responses from five samples. The experiment was replicated three times. Different letters refer to significantly variations between experimental groups (*P* < .05)

**Fig. 6:**
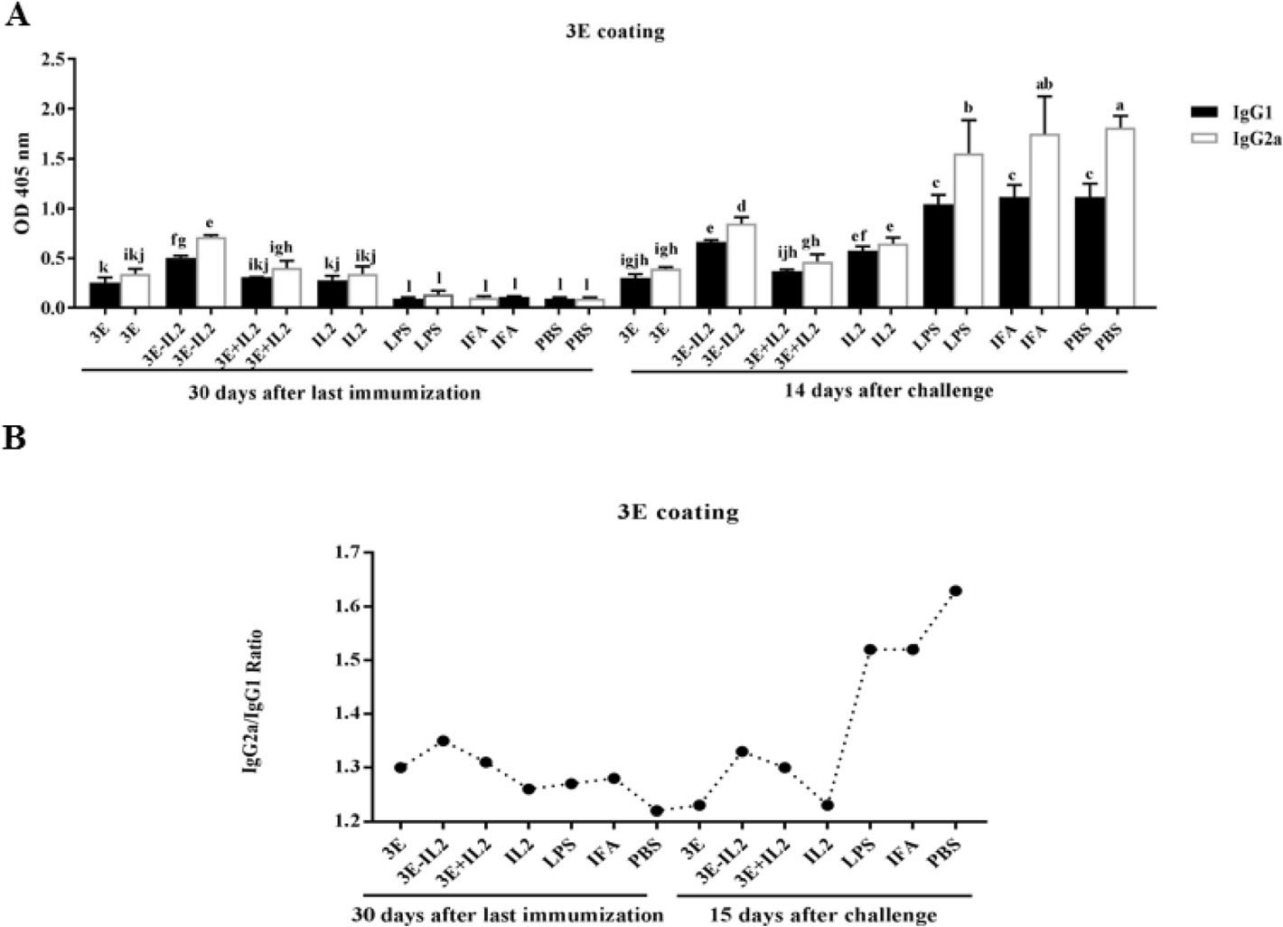
Titer of IgG1 and IgG2a isotypes against 3E protein. **a** titer comparison of IgG1 and IgG2a isotypes sera against 3E protein in 1/50 dilution of sera. Mean comparison of treatments, with significance level of 0.05, were carried out employing Tukey’s test. **b** Ratio of IgG2a to IgG1 in different treatments. Levels of each antibody were measured at OD 405 nm by an ELISA reader. Each value represents the mean of triplicates ± SD of antibody responses from five samples. The experiment was replicated three times. Different letters indicate significant mean difference between experimental groups (*P* < .05)

IgG2a and IgG1 titers were evaluated in sera of vaccinated mice with various formulations containing recombinant protein against Rev1 vaccine (Fig. 4a), the OMP31 antigen (Fig. 5a), and the 3E construct (Fig. 6a).

As can be seen, the titer of isotypes, and especially the IgG2a isotype, were higher in the sera of treated mice after the challenge process, which indicates the efficacy of the vaccination before the challenge (Figs. 4a, 5a and 6a).

In general, before the challenge, all the experimental treatments demonstrated the higher IgG2a and IgG1 levels compared to the negative control groups. But the study on the antibody isotype titer after the challenge indicated the high immunity level at the time of exposure to the acute bacterial strain. As shown in the Figs. 4a, 5a and 6a, treatments which showed high antibody levels before the challenge, were able to produce antibodies with higher potency after the challenge too, which indicated high antibody protection. In negative control treatments that have not received any antigenic vaccine and protein before, the pathogen could engage the immune system. The comparison IgG2a/IgG1 ratio in different treatments has been presented in Figs. 4b, 5b and 6b and it was observed that treatments that were injected with chimeric protein had a higher IgG2a/IgG1 ratio. As it can be seen, the high titer of the antibody, especially IgG2a which is a Th1 index, has been achieved in these treatments.

### Standard tube agglutination test (Wright test)

This test is widely used to detect brucellosis infection. As shown in Table 1 (1 week after the challenge process), positive treatments that had not previously been vaccinated (PBS, IFA, LPS) were considered as sick animal after exposing by acute strain of *B. Melitensis*, because even in the high dilution> 1: 160 of sera, the agglutination process occurred, but in other treatments which received the Rev1 vaccine, or induced by recombinant chimeric proteins (Omp-IL2, 3E-IL2, Omp-IL2 + IFA), the rate of agglutination were < + 2 in less than 1:80 of sera dilution. Therefore, these treatments are not considered as sick.

**Table 1:** The result of the Wight test of blood sera samples after the challenge

	Tteatments												
Concentration of sera	OMP31	IL2	3E	OMP-IL2	OMP+IL2	3E-IL2	3E+IL2	OMP-IL2+ IFA	OMP+ IFA	Rev1	LPS^a^	IFA	PBS
1:10	4+	4+	4+	3+	4+	3+	4+	3+	4+	3+	4+	4+	4+
1:20	4+	4+	4+	2+	3+	2+	4+	2+	4+	2+	4+	4+	4+
1:40	3+	3+	3+	1+	3+	1+	3+	1+	1+	-	4+	4+	4+
1:80	2+	3+	3+	-	1+	-	2+	-	-	-	4+	3+	3+
1:160	-	2+	2+	-	-	-	2+	-	-	-	3+	3+	3+
1:320	-	-	-	-	-	-	-	-	-	-	3+	3+	3+
1:640	-	-	-	-	-	-	-	-	-	-	1+	2+	2+

If all the antigens get agglutinated and the supernatant be clear, the answer is +4

If 75% of antigens get agglutinated and the supernatant be relatively cloudy, the answer is +3

If 50% of antigens get agglutinated and the supernatant be relatively cloudy, the answer is +2

If 25% of antigens get agglutinated and the supernatant be cloudy, the answer is +1

If no sediment is seen and the fluid be completely cloudy, the answer is negative

^a^This treatment contains an extract of the periplasmic part of the BL21 (DE3) bacterium which may contains bacterial lipopolysaccharide (LPS)

### Protection against virulent B. Melitensis

**Fig. 7:**
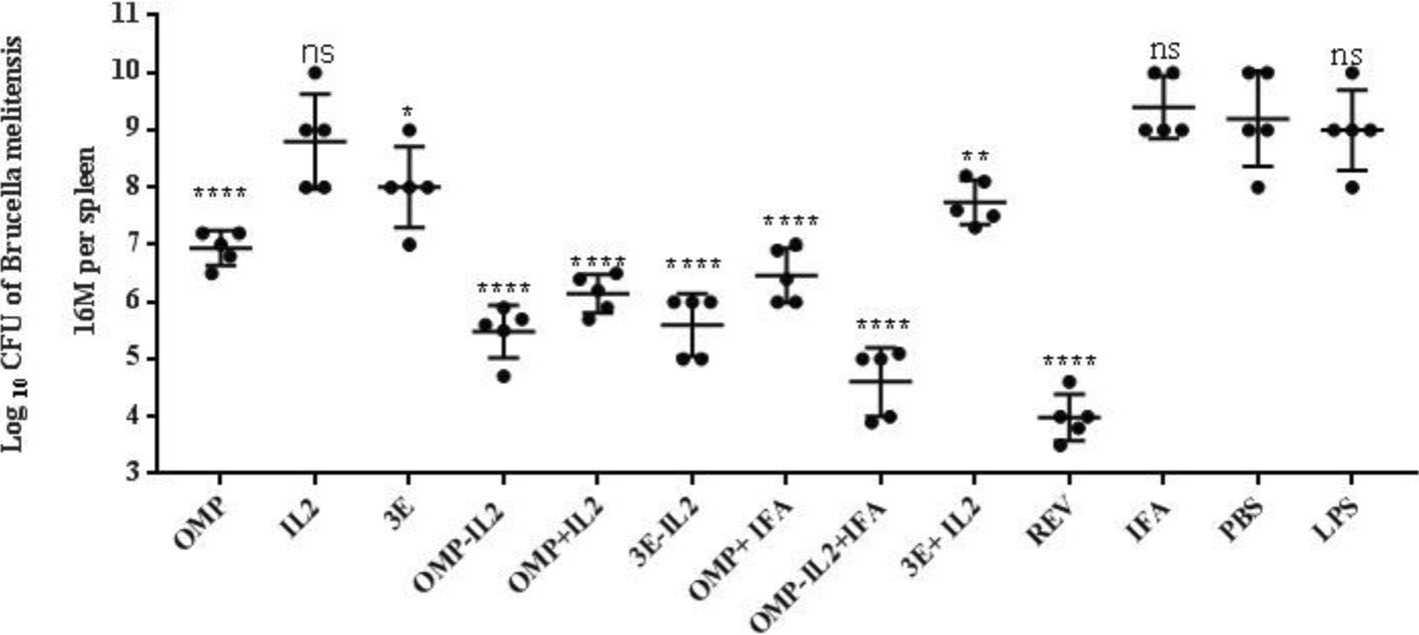
conferred protection of immunized mice with vaccine or recombinant proteins against *B. melitensis M16*. Mean comparison of treatments were carried out employing Tukey’s test with significance level of 0.05. Results are shown as mean ± SD of the log*10*CFU of *B. melitensis M16*per spleen (*n* = 4), **P < 0.01 and ***P < 0.001

As shown in Fig. 7 vaccinated mice with recombinant chimera proteins and *Rev I* vaccine conferred protection against *B. melitensis 16 M* than negative treatments (*p* > 0.005). The number of live bacteria in the spleen was reported as Log_10_ CFU. The highest number of live bacteria in the splenocytes was attributed to negative control treatments that did not receive any vaccine or protein (LPS, PBS and IFA).

## Discussion

Brucella species belong to intracellular pathogens which involves cell mediated immunity. Antigen presenting cells like macrophages process the complex antigens into small peptides and present them through MHC II and MHCI molecules to TCD4+ and cytotoxicTCD8+ respectively [20]. As mentioned before, overcoming the level of IFN-γ in all experimental treatments (with the exception of negative control treatments that did not receive any recombinant protein or vaccine) and, in parallel, high levels of IL-2 emphasizes on the activation of cellular immunity and in particular, Th1 lymphocytes which aimed at the elimination of pathogens using the protective recombinant proteins. These results are in accordance with other studies on the OMP31 antigen or its experimental epitope [8, 21].

IFN-γ by provoking the M1 macrophages [22] increases the MHCII markers and impels them to secrete IL-12 which causes differentiation of Th cells to the Th1 [23]. IFN-γ secretion by Th1 cells also changes the antibody class to IgG class (such as IgG2a) that interferes in complement system fixation and *phagocytosis* [24, 25]. In the present study comparison the IL-4 production, as a biomarker of Th2 lymphocyte activity, confirmed that recombinant chimera effects on strengthening the humoral immunity by Ig switching to IgG and IgE [26]. As it can be traced in Fig. 3, the immune response in all experimental groups against the 3E and OMP31 proteins were similar to response against the Rev1 vaccine. This means that the sequence and structure of the OMP31 antigen and the 3E construct are exactly the same as the *B.melitensis* (Rev1vaccine) vaccine. Therefore, our recombinant proteins are well designed and immunologically bioactive. In this study, in addition to the potency, the efficacy of antigenic constructs was evaluated through the wright test and the microbial load count of the spleen. The results indicated that microbial load in the spleen of the mice receiving recombinant proteins were significantly low. So immunization of them were similar to that of mice received RevI vaccine, which implies to high immunoreaction of the memory cells after the challenge. Low microbial load in the spleen indicates a strong immune response as well as the polarization of the Th1 response against infection of the *B.melitensis*, which is associated with the removal of the intracellular pathogen [26, 27]. On the other hand, cases with the lack of serum agglutination in more than 1/80 dilutions considered as healthy, while the mice in negative treatments (PBS, IFA, LPS) showed ≤ + 2 agglutination in high > 1.80 dilutions.

Referring to the results of cytokines and antibodies, it is worth noting that the treatments with the lowest microbial load of the spleen or the negative wright test showed the highest levels of IFN-γ, IL-2 cytokine and isotype IgG2a. This means that, treating with the recombinant proteins (especially chimera proteins), directly involved cellular immunity through the activation of Th1 cells, and increased the level of IFN-γ secretion, one of the most important macrophage activation factors. Subsequently, activated macrophages (M1) initiate robust oxidative burst activity by phagocyte NADPH oxidase (Nox2) to increase the oxygen free radical (O2−) production and subsequently generating the H2O2, which is associated with the consumption of protons, or reacting with nitric oxide (NO) to create ONOO− that leads to killing of bacterial and the creation of an alkaline environment [28, 29]. Casatara et al., 2007, have studied on the experimental epitope of OMP31 antigens which fused to BLS antigen and proved that this chimera could activate cellular immunity though production the high levels of IFN-γ and IL-2 and IgG2a antibodies, which are cellular immunity indexes. These reports intertwined with the results of the present study.

Since subunit vaccines act to some extent poorly, formulating them into adjuvants can significantly improves their humoral and cellular immune responses [30–32]. Studies have shown that cytokines can improve the ability of vaccines to protect against disease [33]. IL-2 involves as a key role in cascade of events of immune response. This is the first product of activated CD4 + T cells and directly effect on proliferation and differentiation into multiple lineages (Th1/Th2/Treg cells) [34, 35]. In addition, IL-2 interferes in differentiation the nave CD8 + T to effector CTLs and inducing them to expression the IFN-γ [36], TNF-α, and lymphotoxinα [37] to kill infected cells. Therefore, as noted, the presence of IL-2 cytokine, either as chimera or divalent injection, than to the lack of IL-2, drives a protective immunity toward cellular immunity and the IFN-γ and IL-2 production, as expected.

Taken together the results presented in this study showed IL-2 decorated by immunogenic epitopes of OMP31 antigen can confer a significant protective immunity that protects the host against the acute pathogenic strain of Brucella. While, along with the use of molecular adjuvants, the presence of chemical adjuvants further enhances the immune system’s stimulation.

## Conclusions

In this study, OMP31 protein and its immunogenic component, as an experimental epitope with 27 amino acids, were investigated as candidate subunits in experimental vaccines to combat against Brucellosis. Therefore, these two immunogenes were tested in different treatments (3E-IL2, OMP31-IL2, IL2, OMP31 and 3E) on BALB/c mic. The results showed that, rate of IFN-γ and IgG2a isotype, as two major indexes of cellular immunity, in 3E-IL2 treated mice were statistically higher than other treatments (*p* < 0.05). Another phase of this study was investigating the effect of the presence of a chemical adjuvant (IFA) along with a molecular adjuvant (IL2). The results demonstrated that, treated mice with immunogenes which formulated in IFA had highest rate of cytokine and antibody secretion. Also, microbial load count of the spleen and wright test in 3E-IL2 and OMP31-IL2 + IFA treatments, after challenge proses, approved the efficacy of these immunogenic constructs in terms of pathogen removal within 2 weeks after exposure.

## Methods

### Animals

Six to 8 weeks-old female BALB/c mice (purchased from Razi Vaccine and Serum Research institute, agricultural Research Education and Extension Organization (AREEO) and their health status was confirmed by this Research institute) were purchased and randomly divided into thirteen experimental groups in cage contained wood shavings. Mice were housed with appropriate environmental conditions (light (an inverse 12 h day-night cycle with lights on at 8:30 pm), temperature (22 ± 1 °C), humidity (50 ± 5%), and ventilation) and also free access to fresh water and food. Working with animals in study is in agreement with the Ethical Principles for Animal Research established by Ferdowsi university of Mashhad, Mashhad, Iran. And was approved by Biotechnology Laboratory, Department of Animal Science, Faculty of Agricultural, Ferdowsi university of Mashhad, Mashhad, Iran.

### Bacterial strains and vectors

Live attenuated *B. melitensis* (Rev1) bacteria donated from Razi Vaccine and Serum Research institute, Agricultural Research Education and Extension Organization (AREEO). *Escherichia coli* (*E. coli*) DH5α and BL21 (D3) were used as hosts for cloning the recombinant gens and expression the proteins, respectively. Tow pTZ57R/T vectors contained mouse IL-2 gene, OMP31 gene donated by Biotechnology laboratory of Department of Animal Science of Ferdowsi University of Mashhad, whereas coding DNA sequence of 3repeat of experimental epitope region of OMP31 (48–74)_3_ with rigid linker (EAAAK)_2_ was synthesized as inserted in PGH vector (Macrogen, Korea).

### Recombinant proteins production

OMP31-IL2 and 3E-IL2 recombinant proteins were produced as described before [18, 19], briefly rOMP31 (NCBI accession number: KJ193851.1), r3E (OMP31(48–74)3) and *Mus musculus* rIL-2 (NCBI accession number: NM_008366.3) were first amplified individually by flaking primers (containing rigid linker (EAAAK)_2_), then rOMP31 and r3E genes were fused to rIL-2 gene using SOE (Splicing by overhang extension) PCR procedure. Besides, rOMP31, r3E and rIL-2 gens were amplified separately, using specific forward and revers primers without any linkers. These five gens were first T/A cloned and then were sub-cloned into pTZ57R/T and pET-22b (+) vectors respectively using double digestion *(NcoI* and *XhoI* enzymes) and T4 DNA ligase enzyme based on the manufacturer’s instructions kit (Thermo, USA) and followed by transformation into BL21 (D3) bacteria by heat shock method. Recombinant proteins were expressed in 5 h by inducing via 0.5 mM IPTG in 0.06 OD of positive bacteria. PelB signal peptide of pET-22b (+) vectors directs the nascent protein toward the oxidative periplasmic environment. Thus, bacteria were harvested by centrifugation (3000 g, 20 min, 4 °C) and recombinant proteins were extracted using TSE (Tris-HCL200mM pH = 8.0, Sacaros500mM, EDTA1 mM) buffer. These recombinant proteins contain 6-His peptide tag, hence the specific protein purification was done using Ni-NTA affinity chromatography column (Thermo, USA) according to the manufacturer’s protocol. In this term, first of all, samples were dialyzed at 4 °C overnight, then columns were equilibrated and washed twice by cold phosphate buffer saline (PBS), the dialyzed samples contained recombinant proteins were well mixed with nickel-charged affinity resins for 30 min and then were loaded on columns, after by passing the samples, the columns were washed by 20 mM imidazole at 25 °C. Finally, the proteins were eluted from the columns by 4 ml of 250 mM imidazole. Quality of purified protein assessed by SDS-PAGE (12%) and western blotting assay. Their quantity measured by Bradford process using five different dilutions of BSA protein, as the standard proteins and.

### Immunization and experimental treatments

Mice were intraperitoneally immunized three times (days 0, 15 and 30) with 30 μg chimeric proteins (OMP31-IL2 and 3E-IL2) or 30 μg univalent proteins (rOMP31, r3E and rIL2) and/or 30 μg divalent proteins (rOMP31 + rIL2, 3E+ rIL2). One dose of Rev1 vaccine (1–4 × 10^9^ CFU) was used as positive control. Negative controls were injected by Phosphate Buffer Saline (PBS) and Incompletes Fronds Adjuvant (IFA). In this study IL-2 cytokine was used as molecular adjuvant. In order to compare the effect of using the current chemical adjuvants along with molecular adjuvants, additionally, OMP31-IL2 and OMP31 treatments have been considered in two modes: formulated in chemical adjuvant (OMP31-IL2 + IFA and OMP31 + IFA) and without adjuvant (Table 2). To investigate the immune response of lipopolysaccharides (LPS), injection with periplasmic extract of BL21 (D3) bacteria (without any recombinant vector) was considered as another treatments. It should be noted that all studied recombinant proteins were extracted from the periplasmic region of BL21 (D3) bacteria.

**Table 2:** List of treatments and immunization does

	T1	T2	T3	T4	T5	T6	T7	T7	T9	T10	T11	T12	T13
Treatments	OMP31	IL-2	3E	OMP-IL2	OMP+IL2	3E-IL2	3E+IL2	OMP-IL2+ IFA	OMP+ IFA	Rev1	LPS^a^	IFA	PBS
Dose of injection	30μg	30μg	30μg	30μg	30μg+30μg	30μg	30μg+30μg	30μg	30μg	1dose	100μl	100μl	100μl

^a^This treatment contains an extract of the periplasmic part of the BL21 (DE3) bacterium which may contains bacterial lipopolysaccharide (LPS)

### Cytokine detection by ELISA

Spleen cell suspension of immunized mic (5 mice per treatment) prepared by homogenizing the removed spleens in RMPI 1640 medium (supplemented with 4 mM _L_-glutamine, 100u/ml penicillin, 100 μg streptomycin and 10% heat inactivated FBS) 30 days after last immunization. Homogenized suspensions were centrifuged for three mines in 8000 g, deposited cells were dissolved in 1 mL of lysis buffer (0.8% NH4Cl, 2% Tris-base, PH: 7.5) and remained at room temperature for 5 min. 9 ml of the RMPI medium were added and centrifuged again. The supernatant was discarded and the remaining cells were counted after being dissolved in 3 ml of RMPI medium. 4 × 10^6^ splenocyte cells of each treated mouse were cultured in RMPI 1640 medium at 37 °C in 5% CO_2_ using a 96-well flat bottom plate and stimulated *in vitro* with 5 μg/ml of each recombinant proteins. After 48 h, cell culture supernatant was collected and centrifuged at 300 g for 10 mines. IFNγ, IL-2 and IL-4 were measured in spleen cells suspension by sandwich ELISA (sandwich enzyme-linked immunosorbent assay) according to the manufacturer instruction (Mabtech, Nacka, Sweden).

### Evaluation of humoral immune response

To specify humoral immune response against the recombinant proteins, immunized mice were bled 30 days after last immunization (5mic) and 2 weeks after the challenge (5mice). Total IgG antibody of sera was assessed in eight different dilution (1/50–1/64000) to determine the best dilution for measuring the OD using indirect ELISA. In this regard, 1 μg of rOMP31 antigen or r3E peptide or 1× 10^8^ CFU of Rev1 strain of *B.melitensis* were coated in 96-well polystyrene microtiter plate and were incubated for 24 h in 4 °C. Plats were washed three times with PBS containing 0.05% Tween 20 (PBS-T) and blocked for 1 h at 37 °C with 5% skimmed milk in PBS. Then sera (supernatant of centrifuged blood in 3000 g for 20 min) were added serially in diluent buffer containing 0.05% Tween 20 and were incubated for 2 h at room temperature, and were washed three times by PBS-T. 100 μl of 1/10000 dilution of anti-mouse IgG–Horseradish Peroxidase (HRP) conjugate (containing Biotin which connect to coated antigens) antibody (Sigma, USA) was added to each well and plates were incubated for another 2 h at 37 °C. Final washing was done in five times and then 100 μl of 3,3′,5,5′-tetramethyl-benzidine (TMB) substrate (which connect to HRP enzyme) was added to each well and plates were placed in the dark cabin (TMB is light sensitive) for 20–30 min. This reaction was stopped using 2 N H2SO4. Finally, color intensity was measured at OD 450 nm with an ELISA plate reader. Further evaluation was performed on IgG isotypes using 100 μl of 1/50 dilution of sera using IgG1-HRP and IgG2a-HRP conjugated antibodies (Sigma, USA).

### Protection experiment

Four weeks after the last immunization, mice were challenges through intraperitoneal injection of 1 × 10^4^ CFU of *B. melitensis M16* per animal. Two weeks later, infected animals (five mice per treatment) were euthanized by cervical dislocation and their spleen were removed aseptically. Homogenized and lysed splenocyte (as described above) were serially diluted (1/10–1/10^8^). 100 μl of each dilution was spread on Brucella Agar medium (HiMedia, USA) and incubated for 72 h in 37 °C. CFU per spleen was calculated with direct counting of countable dishes from each dilution. Units of protection represented by mean ± SD of log_10_ CFU/spleens of treatments.

### Standard tube agglutination test

Standard tube agglutination test (Wright) is used to confirm the presence of antibody against Brucella in infected or vaccinated animals. One month after the challenge, blood samples were taken from the remaining mice of each treatment (4 mice) and their blood serum were collected. After mixing the sera with the Brucella antigens, due to the specific reaction between the antigen and the antibody (agglutination), a sedimentation occurs at the bottom of the tube which is visible by naked eye. Serial dilution (1:20, 1:40 … 1:640) of sera (collected 2 weeks after challenge) was prepared using physiological saline (5.8 g NaCl and 5 g phenol dissolved in 1litr distilled water and autoclaved for 15 min in 120 °C). Reading agglutination rate was performed after 20 h incubation at 37 °C. If total antigens get agglutinated and the supernatant became clear, or 75% of antigens get agglutinated and the supernatant relatively became cloudy or 50% of antigens get agglutinated and the supernatant became relatively cloudy or 25% of antigens get agglutinated and the supernatant became cloudy the test result will be interpreted as + 4, + 3, + 2 and + 1, respectively, While, no sedimentation and absolute cloudy soluble represents the negative answer.

### Statistical analysis

Analysis of variance (ANOVA) and mean comparison of immune responses data was performed using Tukey’s test and SAS software. GraphPad Prism v6.07 software (GraphPad Software Inc., San Diego, CA, USA) was used to plot the charts for each measured parameter. *P*-values less than 0.05 were considered statistically significant. Values were expressed as mean ± SD.

## Data Availability

The datasets used and/or analyzed during the current study are available from the corresponding author on reasonable request.

## Abbreviations

3E: 3 epitope
ANOVA: Analysis of variance
APCs: antigen-presenting cells
B. melitensis: Brucella melitensis
B. ovis: Brucella ovis
CD8: Cluster of differentiation 8
CFU: Colony-forming unit
CTL: Cytotoxic-T-lymphocyte
DCs: Dendritic cells
ELISA: Enzyme-linked immunosorbent assay
HRP: Horseradish Peroxidase
IFA: Incompletes Fronds Adjuvant
IFN α: Interferon alpha
IFN-γ: Interferon gamma
IgG: Immunoglobulin G
IL2: Interleukin 2
IPTG: Isopropyl β-D-1-thiogalactopyranoside
LPS: Lipopolysaccharides
MΦ: Macrophages
OMP31: 31 KDa outer membrane protein
PBS: Buffer Saline
SDS-PAGE: Sodium dodecyl sulfate–polyacrylamide gel electrophoresis
Th: T helper
TMB: Tetramethyl-benzidine

## References

[1] Pappas G, Akritidis N, Bosilkovski M, Tsianos E (2005). Brucellosis. N Engl J Med.

[2] Brandão AP, Oliveira FS, Carvalho NB, Vieira LQ, Azevedo V, Macedo GC, Oliveira SCJC, Immunology D (2011). Host susceptibility to *Brucella abortus* infection is more pronounced in IFN-γ knockout than IL-12/β2-microglobulin double-deficient mice.

[3] Yang X, Skyberg JA, Cao L, Clapp B, Thornburg T, Pascual DW (2013). Progress in Brucella vaccine development. Front Biol.

[4] Wales JR, Baird MA, Davies NM, Buchan GS (2005). Fusing subunit antigens to interleukin-2 and encapsulating them in liposomes improves their antigenicity but not their protective efficacy. Vaccine.

[5] Liljeqvist S, Ståhl S (1999). Production of recombinant subunit vaccines: protein immunogens, live delivery systems and nucleic acid vaccines. J Biotechnol.

[6] Cassataro J, Velikovsky CA, de la Barrera S, Estein SM, Bruno L, Bowden R, Pasquevich KA, Fossati CA, Giambartolomei GH (2005). A DNA vaccine coding for the Brucella outer membrane protein 31 confers protection against B. melitensis and B. ovis infection by eliciting a specific cytotoxic response. Infect Immun.

[7] Shojaei M, Tahmoorespur M, Soltani M, Sekhavati MH (2018). Immunogenicity evaluation of plasmids encoding Brucella melitensis Omp25 and Omp31 antigens in BALB/c mice. Iran J Basic Med Sci.

[8] Cassataro J, Estein SM, Pasquevich KA, Velikovsky CA, de la Barrera S, Bowden R, Fossati CA, Giambartolomei GH (2005). Vaccination with the recombinant Brucella outer membrane protein 31 or a derived 27-amino-acid synthetic peptide elicits a CD4+ T helper 1 response that protects against Brucella melitensis infection. Infect Immun.

[9] Kaushik P, Chaudhury P, Shukla G, Singh D (2008). Immunogenicity of recombinant Omp28 from Brucella Melitensis in mice. Int J Infect Dis.

[10] Vizcaino N, Cloeckaert A, Zygmunt MS, Dubray G (1996). Cloning, nucleotide sequence, and expression of the Brucella melitensis omp31 gene coding for an immunogenic major outer membrane protein. Infect Immun.

[11] Vizcaíno N, Kittelberger R, Cloeckaert A, Marín CM, Fernández-Lago L (2001). Minor nucleotide substitutions in the omp31 gene ofBrucella ovis result in antigenic differences in the major outer membrane protein that it encodes compared to those of the OtherBrucella species. Infect Immun.

[12] Wang W, Wu J, Qiao J, Weng Y, Zhang H, Liao Q, Qiu J, Chen C, Allain J-P, Li C (2014). Evaluation of humoral and cellular immune responses to BP26 and OMP31 epitopes in the attenuated Brucella melitensis vaccinated sheep. Vaccine.

[13] Cloeckaert A, Jacques I, Grilló MJ, Marín CM, Grayon M, Blasco J-M, Verger J-M (2004). Development and evaluation as vaccines in mice of Brucella melitensis rev. 1 single and double deletion mutants of the bp26 and omp31 genes coding for antigens of diagnostic significance in ovine brucellosis. Vaccine.

[14] Toka FN, Pack CD, Rouse BT (2004). Molecular adjuvants for mucosal immunity. Immunol Rev.

[15] Gutiérrez-Martínez E, Planès R, Anselmi G, Reynolds M, Menezes S, Adiko AC, Saveanu L, Guermonprez P (2015). Cross-presentation of cell-associated antigens by MHC class I in dendritic cell subsets. Front Immunol.

[16] Alberts B, Johnson A, Lewis J, Raff M, Roberts K, Walter P (2002). T cells and MHC proteins.

[17] Siegel JP, Sharon M, Smith PL, Leonard WJ (1987). The IL-2 receptor beta chain-(p70): role in mediating signals for LAK, NK, and proliferative activities. Science.

[18] Nazifi N, Tahmoorespur M, Sekhavati MH, Haghparast A, Behroozikhah MA. Engineering, Cloning and Expression of DNA Sequence Coding of OMP31 Epitope of Brucella melitensis linked to IL-2 in *Escherichia coli*. Int J Infect. 2018;5(3):e68974.

[19] Naghavi M, Sekhavati MH, Tahmoorespur M, Nassiri MR. Design and Production of a Novel Recombinant Chimeric IL2-Omp31 Antigen against Brucella Infection. Arch Razi Instit. 2018;73(3):199–206. 10.22092/ARI.2017.110504.1131 30280839

[20] Mantegazza AR, Magalhaes JG, Amigorena S, Marks MS (2013). Presentation of phagocytosed antigens by MHC class I and II. Traffic (Copenhagen, Denmark).

[21] Yousefi S, Abbassi-Daloii T, Sekhavati MH, Tahmoorespur M (2018). Evaluation of immune responses induced by polymeric OMP25-BLS Brucella antigen. Microb Pathog.

[22] Martinez FO, Helming L, Gordon SJ (2009). Alternative activation of macrophages: an immunologic functional perspective. Annu Rev Immunol.

[23] Smeltz RB, Chen J, Ehrhardt R, Shevach EM (2002). Role of IFN-γ in Th1 Differentiation: IFN-γ Regulates IL-18Rα Expression by Preventing the Negative Effects of IL-4 and by Inducing/Maintaining IL-12 Receptor β2 Expression. J Immunol.

[24] Rogge L (2002). A genomic view of helper T cell subsets. Ann N Y Acad Sci.

[25] Bastos KRB, Barboza R, Sardinha L, Russo M, Alvarez JM, Lima MRDI (2007). Role of endogenous IFN-gamma in macrophage programming induced by IL-12 and IL-18. J Interferon Cytokine Res.

[26] Golding B, Scott DE, Scharf O, Huang L-Y, Zaitseva M, Lapham C, Eller N, Golding HJ (2001). Immunity and protection against *Brucella abortus*. Microbes Infect.

[27] Brandão Ana Paula M. S., Oliveira Fernanda S., Carvalho Natalia B., Vieira Leda Q., Azevedo Vasco, Macedo Gilson C., Oliveira Sergio C. (2012). Host Susceptibility toBrucella abortusInfection Is More Pronounced in IFN-γknockout than IL-12/β2-Microglobulin Double-Deficient Mice. Clinical and Developmental Immunology.

[28] Canton J, Khezri R, Glogauer M, Grinstein S (2014). Contrasting phagosome pH regulation and maturation in human M1 and M2 macrophages. Mol Biol Cell.

[29] El Chemaly A, Nunes P, Jimaja W, Castelbou C, Demaurex N (2014). Hv1 proton channels differentially regulate the pH of neutrophil and macrophage phagosomes by sustaining the production of phagosomal ROS that inhibit the delivery of vacuolar ATPases. J Leukoc Biol.

[30] Finnemann S, Kremsner P, Chaves MF, Schumacher C, Neifer S, Bienzle U (1992). Antibody response inPlasmodium vinckei malaria after treatment with chloroquine and adjuvant interferon-γ. Parasitol Res.

[31] Lucchiari MA, Modolell M, Eichmann K, Pereira CA (1992). In vivo depletion of interferon-gamma leads to susceptibility of A/J mice to mouse hepatitis virus 3 infection. Immunobiology.

[32] Gustafson G, Rhodes MJ (1992). Bacterial cell wall products as adjuvants: early interferon gamma as a marker for adjuvants that enhance protective immunity. Res Immunol.

[33] Nakao M, Hazama M, Mayumi-Aono A, Hinuma S, Fujisawa Y (1994). Immunotherapy of acute and recurrent herpes simplex virus type 2 infection with an adjuvant-free form of recombinant glycoprotein D-interleukin-2 fusion protein. J Infect Dis.

[34] Knoechel B, Lohr J, Kahn E, Bluestone JA, Abbas AK (2005). Sequential development of interleukin 2–dependent effector and regulatory T cells in response to endogenous systemic antigen. J Exp Med.

[35] Wang X, Mosmann T (2001). In vivo priming of CD4 T cells that produce interleukin (IL)-2 but not IL-4 or interferon (IFN)-γ, and can subsequently differentiate into IL-4–or IFN-γ–secreting cells. J Exp Med.

[36] Kasahara T, Hooks J, Dougherty S, Oppenheim JJ (1983). Interleukin 2-mediated immune interferon (IFN-gamma) production by human T cells and T cell subsets. J Immunol.

[37] Lin J-X, Li P, Liu D, Jin HT, He J, Rasheed MAU, Rochman Y, Wang L, Cui K, Liu C (2012). Critical Role of STAT5 transcription factor tetramerization for cytokine responses and normal immune function. Immunity.

